# The Association Between Inflammatory Cell Response and Change in Infarct Size Following STEMI

**DOI:** 10.1016/j.jacadv.2023.100660

**Published:** 2023-10-30

**Authors:** Joyce Lim, Stuart Moir, Nicholas Collins, Sean Hardy, Nishani Mabotuwana, Stuart Sugito, Mohammed Al-Omary, Peter P. Rainer, Andrew J. Boyle

**What is the clinical question being addressed?**

Does the initial inflammatory response affect the change in infarct size post-STEMI treated with primary percutaneous coronary interventions?

**What is the main finding?**Findings from this small study do not suggest a link between a robust early inflammatory response to MI and long-term LV remodeling.

ST-segment elevation myocardial infarction (STEMI) triggers an intense, white cell-mediated inflammatory response proportional to the initial infarct size.^1^, ^2^, ^3^ It remains unclear how the initial leukocyte response, and its differential composition, affects long-term postinfarct left ventricular (LV) remodeling. Therefore, we aimed to investigate the association between leukocytes, neutrophils and monocytes, and change in final infarct size.

This was a retrospective review of a previous clinical trial.^4^ In brief, a phase II randomized double-blinded placebo-controlled study using NP202, a novel oral calcium/calmodulin-dependent protein kinase II delta inhibitor, as an adjunctive therapy for anterior STEMIs. No difference in infarct size or LV remodeling between the 2 groups were found in the original trial so all patients were combined into 1 cohort for analysis. Inclusion criteria for the trial included anterior STEMIs occurring within 5 days, successful primary percutaneous coronary interventions (trials in myocardial infarction flow grade 3 achieved), and a completed postprocedure echo (left ventricular ejection fraction < 45%).

Blood samples were taken at baseline and days 1, 14, and 30 post-STEMI. Serial cardiac magnetic resonance (CMR) studies were completed at each recruitment site and images transmitted to a core lab for blinded analysis, at baseline and 90 days. Study endpoints included change in infarct size from baseline to 90 days using late gadolinium enhancement to assess the degree of irreversible transmural myocardial necrosis. Relative percentage reduction in the infarct size was calculated as the difference between the initial vs 90-day infarct size divided by the initial infarct size. Differences in cell counts over time and change in infarct size between cells, grouped into quartiles based on their peak cell counts, were compared using the Wilcoxon signed-rank test. If significant, prespecified post hoc testing comparing the lowest and highest quartile using the Mann-Whitney test was applied. The association between cell counts and infarct size was analyzed using Spearman’s rank correlation coefficient. Two-sided value of *P* < 0.05 was deemed statistically significant. The study was approved by the Institutional Human Research Ethics Committee at each participating site.

A total of 147 patients (mean age 57.9 ± 10.9 years; 129 men [88%]) were enrolled across 32 sites in the United States, Australia, and New Zealand. Inflammatory markers were available for 143 (97%) patients. Leukocyte counts were highest at admission (median 10.50 × 10^9^ L; IQR: 9.10-12.80) and normalized by day 30 (median 7.40 × 10^9^ L, IQR: 6.48-8.99, *P* < 0.001). Similar trends were observed with neutrophils (median 7.45 × 10^9^ L (IQR: 5.90-9.18) vs 4.6 × 10^9^ L (IQR: 4.00-5.73), *P* < 0.001) and monocytes (median 0.9 × 10^9^ L (IQR: 0.70-1.10) vs 0.6 × 10^9^ L (IQR: 0.50-0.73), *P* < 0.001). Sustained leukocyte and neutrophil response were observed for up to 14 days postinfarct.

The mean initial and 90-day infarct size was 48.2 ± 21.6 g and 30.5 ± 15.3 g, respectively. On average, there was a 28.5% ± 42.6% relative reduction in infarct size over 90 days.

**Figure 1:**
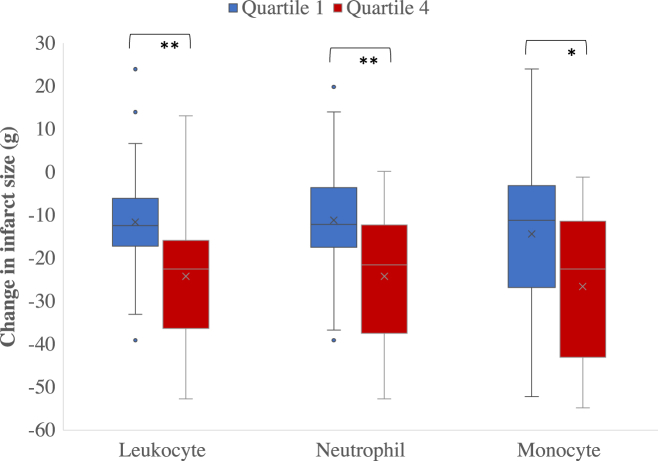
**Box-and-Whisper Plot Showing Change in Infarct Size Stratified by Cell Grouped in Quartiles** Dots represent outliers 1.5 times greater than the interquartile range. ∗*P* < 0.05; ∗∗*P* < 0.01.

Higher peak leukocyte, neutrophil, and monocyte were associated with a greater initial infarct size (leukocyte: *r =* 0.30, *P* = 0.002; neutrophil: *r =* 0.20, *P* = 0.008; monocyte: *r =* 0.20, *P* = 0.012), but not 90-day infarct size (*P* = 0.132, *P* = 0.238, and *P* = 0.228, respectively). Higher peak leukocyte, neutrophil, and monocyte counts were also associated with a greater absolute reduction in infarct size at 90 days (leukocyte: *r* = 0.20, *P* = 0.008; neutrophil: *r* = 0.20, *P* = 0.028; and monocyte: *r* = 0.30, *P =* 0.002). A greater reduction in cell counts between admission to day 30 was associated with a greater reduction in infarct size (leukocyte: *r* = 0.20, *P* = 0.025; neutrophil: *r* = 0.20, *P* = 0.034; and monocyte: *r* = 0.20, *P =* 0.037). After grouping the leukocytes, neutrophils, and monocytes into their respective quartiles, between group differences were observed in the change in infarct size over 3 months (leukocyte: *P* < 0.003; neutrophil: *P* = 0.008; monocyte: *P* = 0.026) (Figure 1). However, there was no apparent association between relative percentage reduction and peak cell counts (leukocyte: *P* = 0.290; neutrophil: *P* = 0.209; and monocyte: *P* = 0.118) or differences between admission to day 30 cell counts (leukocyte: *P* = 0.082; neutrophil: *P* = 0.608; and monocyte: *P* = 0.342).

Our study, therefore, suggests that while higher initial peak cell counts are associated with a greater absolute reduction in infarct size at 3 months, it is not linked with the relative percentage reduction in infarct size for individual patients. We note there was only a weak correlation between peak cell counts and absolute reduction in final infarct size. Findings from this small study do not suggest a link between peak cell counts and long-term LV remodeling.

This study is limited to a post hoc analysis of anterior STEMIs, only. The analysis was limited by the small sample size, differences in patient characteristics not being accounted for, and lack of control over the type I error rate despite multiple comparison included into the analysis. Baseline CMR images were acquired anytime between 48 hours to 5 days post-MI, potentially introducing significant differences in the infarct size as a product of time post-MI.^5^ Future studies addressing such limitations are therefore required to examine the association between STEMIs involving all coronary territories and CMR-derived infarct size. At present, however, our preliminary study suggests that a robust early inflammatory response to MI is not associated with long term LV remodeling.
